# Corrigendum: Improved diagnosis of inflammatory bowel disease and prediction and monitoring of response to anti-TNF alpha treatment based on measurement of signal transduction pathway activity

**DOI:** 10.3389/fphar.2023.1183639

**Published:** 2023-04-05

**Authors:** Wilbert Bouwman, Wim Verhaegh, Anja van de Stolpe

**Affiliations:** Philips Research, Eindhoven, Netherlands

**Keywords:** ulcerative colitis, crohn’s disease, IBD, signal transduction pathway, treatment response

In the published article, there was an error in Figure 2 as published. In this figure for the prediction and assessment of response to anti-TNFα remission-induction treatment in CD (A) a different TGFbeta pathway model version was used**.** The corrected Figure 2 and its caption appear below.

**FIGURE 2:**
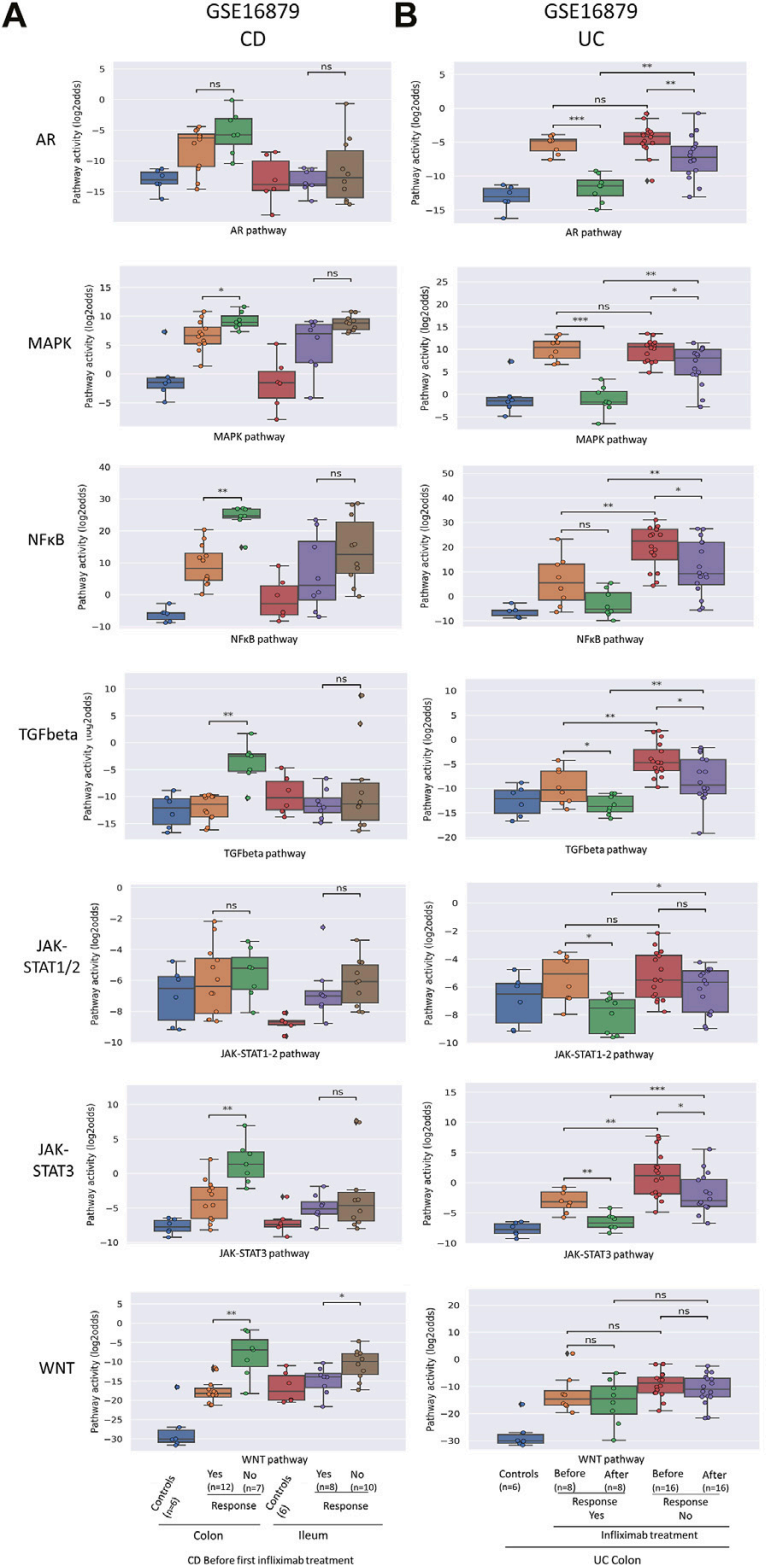
Prediction and assessment of response to anti-TNFα remission-induction treatment in CD **(A)** and UC **(B)**. Datasets GSE16879. STP activity analysis of intestinal mucosa samples for prediction of response to anti-TNFα remission induction treatment in patients with CD and UC. STP PAS before and after remission induction treatment. For details, see Methods. STP PAS are shown for the AR, MAPK, NFκB, TGFβ, JAK-STAT1/2, JAK-STAT3, and Wnt STPs. Two sided Mann–Whitney–Wilcoxon statistical tests were performed; p-values are indicated in the figures as *p < 0.05, **p < 0.01, ***p < 0.001, ****p < 0.0001, ns: not significant. For supporting analysis results, see Supplementary Table S1.

The authors apologize for this error and state that this does not change the scientific conclusions of the article in any way. The original article has been updated.

