# Supplementary material for: Improved diagnosis of inflammatory bowel disease and prediction and monitoring of response to anti-TNF alpha treatment based on measurement of signal transduction pathway activity
Source: Front Pharmacol. 2022 Oct 13;13:1008976. doi: 10.3389/fphar.2022.1008976 (PMC10115426; doi:10.3389/fphar.2022.1008976)
Supplement: Supplementary file 2 [file DataSheet1.docx]

**Improved diagnosis of inflammatory bowel disease and prediction and monitoring of response to anti-TNF alpha treatment based on measurement of signal transduction pathway activity.**

Wilbert Bouwman, Wim Verhaegh, Anja van de Stolpe

Philips Research, High tech campus 34, Eindhoven, The Netherlands

Corresponding author: Wilbert Bouwman, [Wilbert.Bouwman@philips.com](mailto:Wilbert.Bouwman@philips.com)

**Conflict of interest statement: All authors are regular employees of Philips**


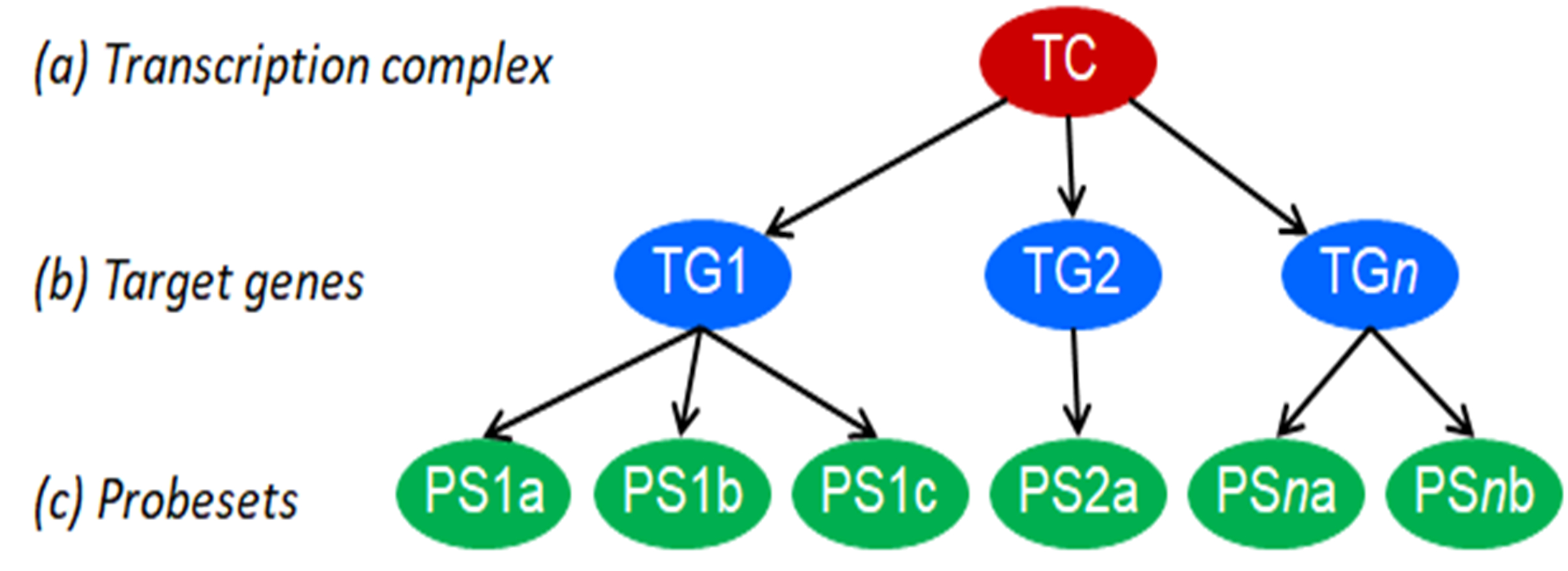


Figure S1. **Knowledge-based Bayesian computational signal transduction pathway model.** The network structure is used as basis for our modeling approach shown as a simple model of the transcriptional program of a cellular signal transduction pathway, consisting of three types of nodes: a) transcription factor complex (TC), b) target gene (TG), and c) microarray probe sets (PS) corresponding to target genes.


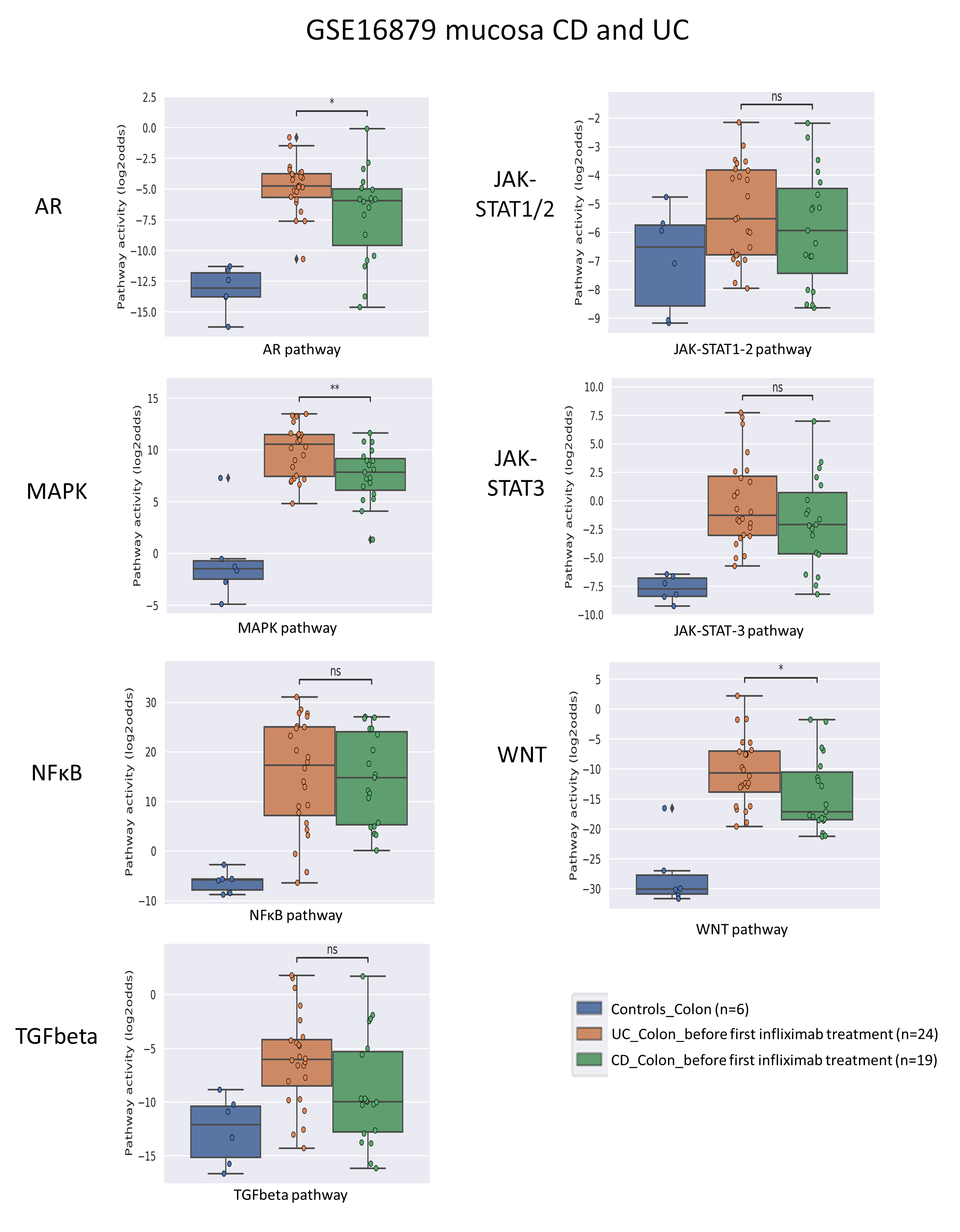


Figure S2. **Differential diagnosis of CD and UC.** Dataset GSE16879. Illustration of differences in STP PAS between CD and UC, irrespective of response to anti-TNFα treatment. Mucosa samples are from healthy control individuals and pre-treatment mucosa samples from IBD patients. For details, see Methods. Signal transduction pathway (STP) activity scores (PAS) are shown (A-G) for the androgen receptor (AR), MAPK , NFκB, TGFβ, JAK-STAT1/2 (STAT1-2), JAK-STAT3 (STAT3), and Wnt STPs. PAS on Y-axis on a log2 odds scale. From left to right: Healthy control, CD, UC. Two sided Mann-Whitney-Wilcoxon statistical tests were performed; p-values are indicated in the figures as *p < 0.05, **p < 0.01, ns: not significant.


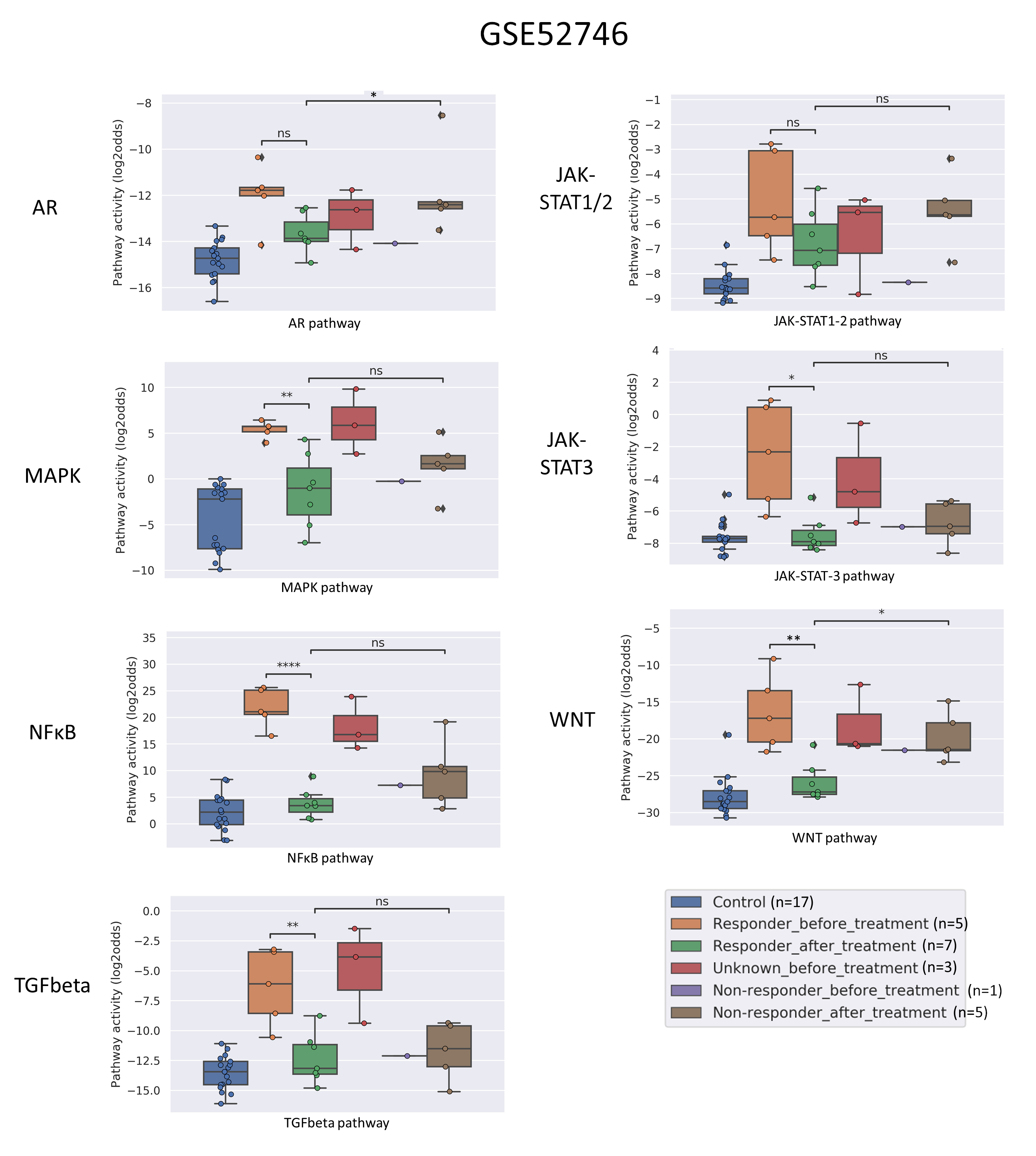


Figure S3: **Prediction and assessment of response to anti-TNFα remission-induction treatment in CD.** Dataset GSE52746 on top. STP activity analysis of intestinal mucosa samples for prediction of response to anti-TNFα remission induction treatment in patients with CD. STP PAS before and after remission induction treatment, for responder and non-responder patients. For some patients only a sample before treatment was taken. For details, see Methods. STP PAS are shown for the androgen receptor (AR), MAPK , NFκB, TGFβ, JAK-STAT1/2 (STAT1-2), JAK-STAT3 (STAT3), and Wnt STPs. PAS on Y-axis on a log2 odds scale. From left to right: Dataset GSE52746: Healthy control, pretreatment samples, response unknown; responders before and after treatment; non-responders before and after treatment; dataset Two sided T-test statistical tests were performed; p-values are indicated in the figures as *p < 0.05, **p < 0.01, ****p < 0.0001, ns: not significant.
